# Orthopedic Surgery Residency Program Rankings and Gender Diversity

**DOI:** 10.7759/cureus.56365

**Published:** 2024-03-18

**Authors:** Yuri Han, Lilah Fones, Rachel Shakked, Sommer Hammoud

**Affiliations:** 1 Orthopedic Surgery, Robert Wood Johnson Medical School, Piscataway, USA; 2 Orthopaedic Surgery, Rothman Orthopaedic Institute at Thomas Jefferson University, Philadelphia, USA

**Keywords:** orthopedic surgery, doximity, residency program director, residency program, gender disparities

## Abstract

Background

Orthopedic surgery residency programs are some of the least gender-diverse specialty programs in medicine. Despite strong representation at the undergraduate and medical school levels and increased applications to orthopedic surgery residency programs by women, there is still a substantial gender gap at the resident level. This study explores the relationship between the gender diversity of orthopedic surgery residency programs and program rankings.

Methodology

Program rank, program director gender identity, and gender diversity data were collected for the top 100 programs by reputation in Doximity. Gender diversity was measured as the proportion of female residents in the program and alumni.

Results

The greatest percentage of women in a program was 33% and the smallest was 3%. After linear regression analysis, we found that there was a statistically significant positive correlation between program rank and the proportion of women. The higher ranked a program was, the greater the proportion of women. There was no significant correlation between program director gender, appointment year, and program rank.

Conclusions

These results suggest that, although there is still a long way to go before closing the gender gap in orthopedic surgery residency programs, higher-ranked programs are associated with greater gender diversity than their lower-ranked counterparts.

## Introduction

The field of orthopedic surgery is a male-dominated surgical specialty. Despite that more than half of medical students (50.5%) in the United States are female, and more female medical students are graduating every year [1], orthopedic surgery remains predominantly male. As of 2018, only 5.3% of orthopedic surgeons were women [2]. Compared to other surgical specialties, orthopedic surgery has seen a slower rate of pursuit among female medical students [3,4]. Although Onuoha et al. showed that the proportion of female orthopedic surgery residents increased by 24% between 2007 and 2019 [5], it remained the smallest proportion of women compared to the other surgical specialties, including neurosurgery and general surgery.

Similarly, leadership in orthopedic surgery is predominantly male. As of 2019, 19.9% of orthopaedic surgery faculty were female, and, in 2020, 9% of program directors were female [6]. Orthopedic surgery had the second lowest proportion of female program directors behind neurosurgery. However, there was greater female representation in academics when compared to private practice, further stressing the importance of academic institutions in promoting gender diversity [7].

Academic institutions foster gender diversity in orthopedics through outreach and early exposure. For instance, multiple organizations, including the Ruth Jackson Orthopaedic Society, Nth Dimensions, and the Perry Initiative, emphasize the importance of early exposure and mentorship in promoting interest in orthopedic surgery among female medical students [8]. Such outreach programs have strong ties with academic institutions and, thus, create a pipeline for female medical students interested in orthopedic surgery. Similarly, more women were likely to apply to orthopedics residency when attending medical school with greater gender diversity among the orthopedic faculty [9]. Another factor female medical students consider when applying to orthopedic residency programs is academic prestige [10]. One proposed objective measure of academic success is the Hirschberg index, a metric for publication impact, and editorial board positions of top journals, which can provide a picture of a program’s academic contribution to the field [10]. Alternatively, Doximity published an annual orthopedic residency program ranking that many medical students utilize to initially learn about residency programs and assist in creating their rank lists [11,12].

The primary purpose of our study is to investigate the relationship between orthopedic surgery residency program rankings and gender diversity using Doximity’s rank and gender diversity data. We hypothesized that higher-ranked programs would have greater gender diversity compared to lower-ranked programs. Our secondary aim was to evaluate the representation of female program directors based on program ranking.

## Materials and methods

The top 100 orthopedic surgery residency programs as measured by reputation according to Doximity were extracted from Doximity.com on August 9th, 2023 [13]. In Doximity, programs are ranked from one (highest-ranked program) to 209 (lowest-ranked program). Doximity reputation data is measured by a nomination system where eligible Doximity physicians can submit nominations for up to five residency programs that offer the best clinical training, and the data is pooled from the previous three years to create annually published residency program rankings [11].

Doximity also presents the proportion of female residents in each program as “Gender Balance” via a visual chart with percentages of female and male residents of both current and recently graduated residents [13]. For each of the 100 top orthopedic residency programs included in this study, the percentage of female residents was extracted from the Doximity website.

The current program director’s gender and year of appointment were also collected on each program’s Doximity page [13]. The program director’s gender was determined by pronoun preference on Doximity first; if pronouns were not found on Doximity, press mentions were used through Google search.

Linear regression was performed in R to examine the relationship between program rank and the proportion of women. Secondary analysis via logistic regression was performed to examine the relationship between the program director’s gender and both program rank and year of appointment. Descriptive statistics were also performed. Categorical data is presented as count (percentage) and continuous data is presented as mean (standard deviation (SD)).

## Results

Of the top 100 residency programs, four did not have diversity data on Doximity. These programs were excluded. The average proportion of women in a program was 14% (SD = 6.36%). The greatest proportion was 33% and the smallest was 3%.

**Figure 1:**
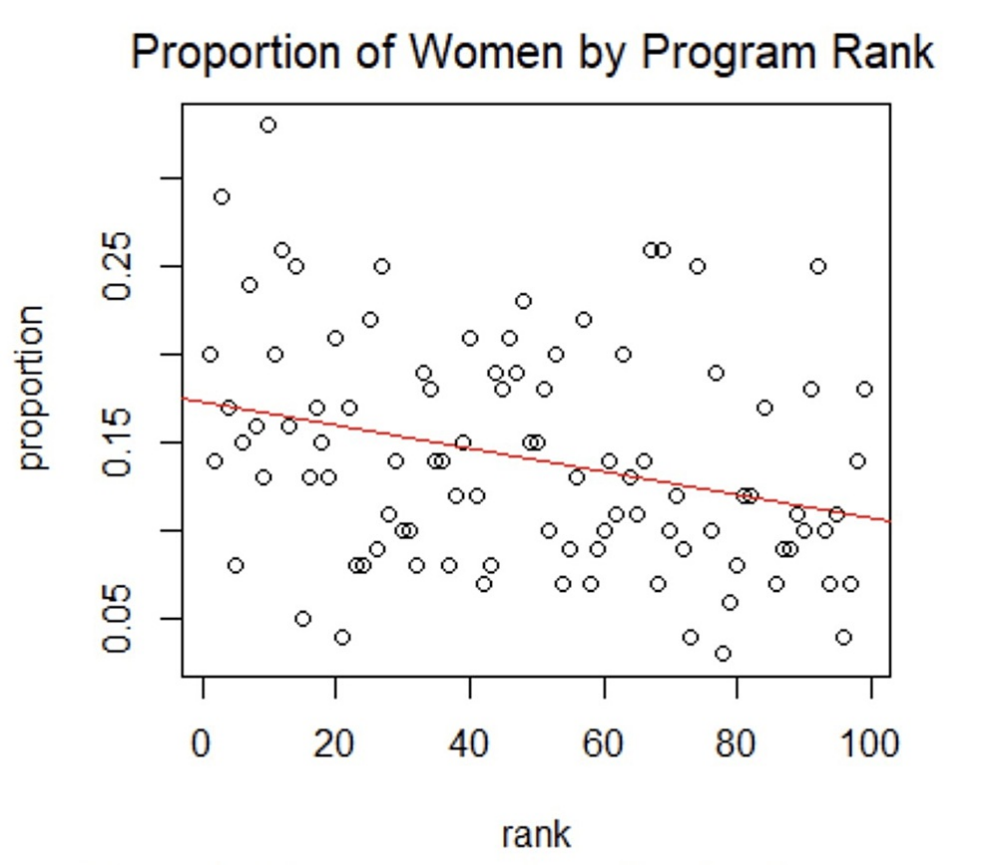
Linear regression plot for the proportion of women in a residency program by program ranking.

After linear regression analysis (Figure 1), a statistically significant correlation was found between program rank and the proportion of women. The linear regression coefficient was found to be -0.000654 (SD = 0.000219). According to Akoglu’s guide to correlation coefficients, this correlation, although statistically significant, is considered a weak one [14]. Our results show that, with a correlation coefficient of -0.000654, the higher the program rank (closer to one), the greater the proportion of women (p < 0.05).

**Figure 2:**
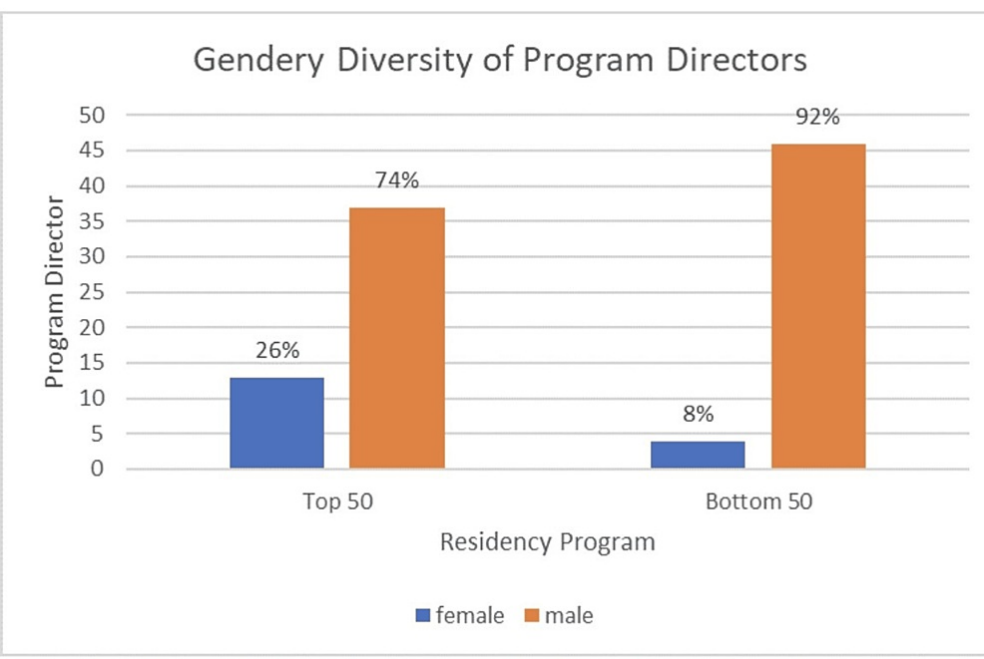
Gender diversity of program directors of the top 50 and bottom 50 ranked programs.

Of the top 100 orthopedic residency program directors, 17 were female (17%) and 83 (83%) were male. When split into the top 50% and bottom 50%, there were more female program directors in the top 50% (Figure 2). There was no statistically significant correlation between the program directors’ gender and program rank. When examining the program directors’ gender and year of appointment, the year of appointment for female program directors was on average 2018 (SD = 4.70), relative to 2015 (SD = 5.56) for their male counterparts. However, there was no statistically significant relationship between the directors’ gender and year of appointment.

## Discussion

Previous reports of gender diversity in science, technology, engineering, and mathematics (STEM) fields by Su et al. have highlighted that, while the relationship between academics and diversity is multifaceted and nuanced, it is likely that department rankings and prestige level may play some role in promoting gender diversity [15]. Here, we report a statistically significant correlation between program rank and gender diversity. It can be hypothesized that higher-ranked programs are more likely to have access to more resources and a greater capacity to host outreach programs aimed at promoting diversity in the field, thus attracting more female medical students. Female medical students applying to orthopedic surgery residencies may also be drawn to such programs due to such programs’ commitment to gender diversity [9]. Indeed, in a 5-year update and comparison of factors influencing female representation in orthopedic surgery residency programs by Julian et al., it was found that there are significantly higher percentages of female residents in residency programs with higher percentages of female faculty [16]. Our results may also suggest differences in residency program cultures regarding gender diversity and may reflect biases that exist at higher versus lower-ranked programs, which may influence match lists created by program directors and faculty.

While we hypothesized that higher-ranked programs would have greater diversity in program directorship and that female directorship would have more recent appointments, we found that neither relationship was of statistical significance. Meadows et al. found that there was an increase in gender diversity among chairpersons from 2007 to 2019/2020, but no change in the diversity of program directors, with only 9% of program directors being female in 2020 [6]. In our study, we found that 17% of the program directors were female. It is important to note that we only examined the current gender diversity among program directorships. Future directions of study should include a comparative analysis over several years to examine trends in gender diversity among program directors in orthopedic surgery.

In a more recent study, Elkadi et al. found that 11.6% of program directors were female as of 2022 [17], which contrasts the results of our study of 17% of program directorship being female and the results of the Meadows et al. study of 9% [6]. While this suggests an increase in female program directorship from 2020 to 2020, it is less than the percentage obtained in our investigation. We examined program directors from the top 100 programs based on reputation, while Elkadi et al. assessed those from 200 orthopedic residency programs, suggesting increased female representation in program directorship at higher-ranked programs [17]. Indeed, a paper investigating the characteristics of leadership in “top-tier” orthopedic surgery residency programs supports this, as they found that higher-tier programs had a larger proportion of women leaders with statistical significance after examining 193 programs [18]. Therefore, while our study did not find a significant difference between the proportion of women in program directorship of the top and bottom 50 of the programs we included, it is likely that there will be a significantly higher proportion of female program directors in the top 100 programs we included vs. the bottom 100. Furthermore, given the results of our study, the Julian et al. five-year update, and the Huyke-Hernandez et al. “top-tier” program analysis, future studies can hypothesize that the faculty of higher-ranked programs are more gender diverse, which could correlate with the higher proportion of female leaders [18] and residents (our present study) at higher-ranked programs.

This study has several limitations. Regarding program rankings, this study relied on Doximity’s rank list according to their measurement of reputation. According to Doximity, reputation data was measured by a nomination system: eligible Doximity physicians can submit nominations for up to five residency programs that offer the best clinical training, and this data is pooled from the previous three years [13]. Given this, our results are subject to bias, as rankings were determined exclusively by Doximity members. Additionally, gender diversity data was also determined by Doximity by using the profiles of current and recent alumni residents. For instance, when comparing the number of current residents listed on the HSS residency program’s website and the number of current residents listed on Doximity’s page for the HSS program (as of October 27, 2023), the HSS website lists 46 current residents, while Doximity lists 36. Therefore, our gender data results are also subject to bias, as only Doximity members were included. This study also only examined gender diversity using the designations “man/woman” and “male/female.” Thus, our results do not reflect the full breadth of gender diversity of orthopedic surgery residency programs, such as residents who identify as gender-fluid and non-binary. When surveyed about the inclusivity of different surgical specialties, 84% of LGBTQ+ medical students selected orthopedic surgery as a specialty that is not welcoming. Orthopedic surgery was the most common response followed by general surgery and neurosurgery [19]. These survey results emphasize the need for greater consideration of gender identity in the field of orthopedics and in the promotion of gender equality in the field.

Another limitation of this study is that it did not analyze diversity in orthopedics regarding ethnic or racial diversity, orthopedics being one of the least diverse in this regard as well, with only 20.5% minority representation among program directors and only 4.1% and 2.7% African American and Hispanic orthopedic trainees, respectively [6,20].

Our study’s strengths include exploring the relationship between program rank and gender diversity of orthopedic surgery residency programs, a relationship that has never been directly studied in previous work on gender diversity in orthopedics. Doximity’s program rank and gender diversity features provided readily standardized access to such measures upon data collection despite the aforementioned limitations.

## Conclusions

Our study suggests that higher-ranked orthopedic surgery residency programs are more gender diverse. While higher-ranked programs are associated with a greater proportion of women in programs, there was no significant relationship between program ranking and gender diversity of program directorship. These results suggest that factors affecting program rank and reputation may play a role in promoting gender diversity in orthopedic surgery among applicants to residencies, highlighting the importance of academic programs in promoting a gender-diverse culture in the field and closing the orthopedic gender gap.
